# Affective temperament and emotion regulation as predictors of clinical outcomes in women with idiopathic granulomatous mastitis

**DOI:** 10.1590/1806-9282.20260376

**Published:** 2026-08-07

**Authors:** Cagri Akalın, Ilker Sengul, Demet Sengul, Halil Ibrahim Tas, Gabriela Bezerra Nóbrega, Cecília da Silva Ferreira, Isaque da Silva Ferreira, José Maria Soares

**Affiliations:** 1 Ordu University, Faculty of Medicine, Department of General Surgery – Ordu, Turkey.; 2 Giresun University, Faculty of Medicine, Thyroidology Unit – Giresun, Turkey.; 3 Giresun University, Faculty of Medicine, Division of Endocrine Surgery – Giresun, Turkey.; 4 Giresun University, Faculty of Medicine, Department of General Surgery – Giresun, Turkey.; 5 Giresun University, Faculty of Medicine, Department of Pathology – Giresun, Turkey.; 6 Samsun University, Faculty of Medicine, Department of Psychiatry – Samsun, Turkey.; 7 Universidade de São Paulo, Hospital das Clínicas, Faculdade de Medicina, Departamento de Obstetrícia e Ginecologia, Disciplina de Ginecologia, Laboratório de Ginecologia Estrutural e Molecular – São Paulo (SP), Brazil.

**Keywords:** Neuroendocrinology, Emotional regulation, Granulomatous mastitis, Human papillomavirus infections, Breast, Pathology

## Abstract

**OBJECTIVE:**

The aim of this study was to evaluate affective temperament, emotion regulation, and anxiety–depression symptoms in women with idiopathic granulomatous mastitis, and to determine whether these psychological factors predict clinically relevant outcomes.

**METHODS:**

This case–control study included 97 patients with idiopathic granulomatous mastitis and 100 healthy controls. Affective temperament, emotion regulation, and anxiety–depression symptoms were assessed using the Temperament Evaluation of Memphis, Pisa, Paris, and San Diego Autoquestionnaire, the Difficulties in Emotion Regulation Scale, and the Hospital Anxiety and Depression Scale, respectively. Multivariable logistic regression analyses had been performed to identify psychological predictors of recurrence, treatment resistance, and fistula formation.

**RESULTS:**

Between-group analyses showed higher depressive and anxious temperament traits in women with idiopathic granulomatous mastitis, greater emotion regulation difficulties, and elevated anxiety–depression symptoms (all p<0.001). Emotion regulation impairment independently predicted treatment resistance (OR 2.86, p=0.004). In contrast, anxious temperament was inversely associated with both recurrence (OR 0.33, p=0.012) and treatment resistance (OR 0.35, p=0.010). No psychological variable predicted fistula formation.

**CONCLUSION:**

Idiopathic granulomatous mastitis is associated with a distinct psychosomatic vulnerability profile. While impaired emotion regulation increases the risk of refractory disease, anxious temperament may be linked to protective vigilance-related behaviors. These findings support the integration of psychoneuroimmunology-informed psychological assessment into idiopathic granulomatous mastitis management.

## INTRODUCTION

Idiopathic granulomatous mastitis (IGM) is a chronic, relapsing inflammatory breast disease that predominantly affects young women of reproductive age. Histopathologically, it is characterized by non-caseating granulomas, recurrent abscesses, and occasional fistula formation. Although IGM is a benign condition, its clinical course is frequently complicated by recurrence or treatment resistance, resulting in a substantial physical and psychological burden for affected patients^1,2,3,4,5^.

The aetiopathogenesis of IGM has not yet been fully elucidated. Autoimmune mechanisms, hormonal influences, and infectious agents—particularly Corynebacterium kroppenstedtii—have been implicated; however, these biological factors alone are insufficient to explain the marked heterogeneity observed in disease severity, treatment response, and recurrence rates, which variability suggests that additional modifying factors may contribute to the clinical course of the disease^2,3,4,5^. Psychoneuroimmunology predicts that stable psychological traits influence immune and inflammatory processes. Affective temperament traits, such as depressive and anxious dispositions, as well as difficulties in emotion regulation, have been associated with alterations in autonomic nervous system regulation, hypothalamic–pituitary–adrenal (HPA) axis responsiveness, glucocorticoid receptor sensitivity, and pro-inflammatory cytokine signalling^6-9^, which mechanisms may contribute to the persistence of inflammatory activity and reduced responsiveness to immunosuppressive therapies^9,10,11^.

In several chronic inflammatory diseases, including inflammatory bowel disease, rheumatoid arthritis, and psoriasis, psychological factors have been associated with disease activity and treatment outcomes^10,11,12^. In contrast, evidence regarding psychosomatic determinants in IGM remains limited. Although increased anxiety and depressive symptoms have been reported in women with IGM^13^, the roles of affective temperament structure and emotion regulation capacity in predicting clinically meaningful outcomes—such as recurrence and treatment resistance—have not been systematically investigated.

Accordingly, the aim of this study was to compare affective temperament traits, emotion regulation difficulties, and anxiety–depression symptoms between women with IGM and healthy controls, and to evaluate whether these psychological dimensions predict key clinical outcomes, including recurrence, treatment resistance, and fistula formation.

## METHODS

### Study design

This case–control study was conducted at the Department of General Surgery, Ordu University Faculty of Medicine, Ordu, Turkey, between January 2023 and November 2025. A total of 97 women with histopathologically confirmed IGM were consecutively recruited. The control group consisted of 100 female volunteers without a history of breast disease or chronic inflammatory conditions. The diagnosis of IGM was established based on compatible clinical presentation, radiological findings (breast ultrasonography and/or magnetic resonance imaging), and histopathological confirmation obtained by core-needle or excisional biopsy demonstrating non-caseating granulomatous inflammation^1,2,3,4^.

Inclusion criteria for the IGM group were age between 18 and 55 years, histopathological confirmation of IGM, and availability of complete clinical and psychometric data. Exclusion criteria included pregnancy or lactation, known autoimmune disease, current or recent (within 6 months) use of psychiatric medication, breast malignancy, and incomplete psychological assessments. The same exclusion criteria were applied to the control group. All the participants provided written informed consent prior to enrolment. The study protocol was conducted in accordance with the principles of the Declaration of Helsinki.

### Clinical variables and outcome definitions

The sociodemographic and obstetric characteristics had been collected using structured interviews and medical record review to assess baseline comparability between groups. The following

IGM-related clinical outcomes were prospectively defined: (i) Recurrence: development of a new lesion, abscess, or fistula occurring ≥6 months after documented clinical and radiological remission, (ii) Treatment resistance: absence of measurable clinical and/or radiological improvement within 12 weeks of corticosteroid therapy, with or without additional immunosuppressive agents, (iii) Fistula formation: occurrence of a sinus tract or fistula during or after treatment, and (iv) Disease duration: time interval (in months) between symptom onset and histopathological diagnosis.

### Psychological and psychoneuroendocrine assessments

Psychometric evaluations had been performed after histopathological confirmation of IGM and before initiation of immunosuppressive therapy for this disease.

### Affective temperament

Affective temperament traits were assessed using the validated Turkish version of the Temperament Evaluation of Memphis, Pisa, Paris, and San Diego Autoquestionnaire (TEMPS-A), which evaluates depressive, anxious, cyclothymic, hyperthymic, and irritable temperament dimensions^6,7,8^. Dominant temperament was defined as the temperament subtype with the highest standardized subscale score.

### Emotion regulation

Difficulties in emotion regulation were measured using the 16-item Difficulties in Emotion Regulation Scale (DERS-16), validated in the Turkish population^9,10,11^. Higher total scores indicate greater impairment in emotion regulation capacity.

### Anxiety and depression symptoms

Current anxiety and depressive symptoms were evaluated using the Hospital Anxiety and Depression Scale (HADS). Anxiety (HADS-A) and depression (HADS-D) subscale scores were analyzed as continuous variables and categorized for regression analyses using standard cut-off values (0–7: normal; 8–21: borderline or clinical case)^10,11,12^. Ultimately, all the psychometric instruments demonstrated good internal consistency (Cronbach’s α=0.82–0.94).

### Statistical analysis

The statistical analyses were performed using International Business Machines Corporation SPSS Statistics version 28.0 (IBM Corp., Armonk, NY, USA), and the data distribution was assessed using the Shapiro-Wilk test. Secondly, the continuous variables were compared between groups using independent-samples t-tests, and categorical variables were analyzed using χ^2^ tests. Afterward, the effect sizes were calculated using Cohen’s d for continuous variables and Cramér’s V for categorical comparisons. To identify psychological predictors of recurrence, treatment resistance, and fistula formation, multivariable binary logistic regression analyses were conducted. Of note, variables with p<0.10 in univariable analyses were entered into multivariable models alongside theoretically prioritized affective temperament variables. Last but not least, the events-per-variable ratios were sufficient to minimize model overfitting. The model fit was evaluated using the -2 log-likelihood statistic, Nagelkerke R^2^, and the Hosmer-Lemeshow goodness-of-fit test. All the tests were two-tailed, and p<0.05 were considered statistically significant.

## RESULTS

### Participant characteristics

A total of 197 women were included in the analysis, comprising 97 cases with IGM and 100 controls. No significant between-group differences were observed in age, body mass index, education level, marital status, smoking status, parity, or breastfeeding history (all p>0.05), indicating adequate baseline comparability between the groups (Table 1).

**Table 1:** Sociodemographic and clinical characteristics.

Variables	IGM group (n=97)	Control group (n=100)	p-value
Age (years), mean±SD	35.7±3.5	34.2±5.4	0.34
Body mass index (kg/m^2^), mean±SD	27.2±1.9	26.9±2.3	0.74
Education, n (%)			
Primary school	0 (0%)	1 (1%)	0.44
Middle school	26 (26.8%)	21 (21%)	
High school	45 (46.4%)	45 (45%)	
University or higher	26 (26.8%)	33 (33%)	
Marital status, n (%)			
Single	11 (11.3%)	10 (10%)	0.72
Married	78 (80.4%)	85 (85%)	
Divorced	6 (6.2%)	3 (3%)	
Widowed	2 (2.1%)	2 (2%)	
Smoking, n (%)	24 (24.7%)	20 (20%)	0.49
Parity ≥1, n (%)	77 (79.4%)	82 (82%)	0.64
History of breastfeeding, n (%)	58 (59.8%)	72 (72%)	0.74

Values are presented as mean±SD or n (%). BMI: body mass index; IGM: idiopathic granulomatous mastitis; SD: standard deviation. An independent-samples t-test was used for continuous variables and a χ^2^ test for categorical variables.

### Psychological characteristics: between-group comparisons

The women with IGM showed significantly higher scores across multiple psychological dimensions than healthy controls (Table 2). Specifically, depressive temperament (TEMPS-dep), anxious temperament (TEMPS-anx), cyclothymic temperament, and irritable temperament scores were significantly elevated in the IGM group (all p<0.001). In contrast, the hyperthymic temperament scores were significantly lower among women with IGM (p<0.001). The women with IGM also exhibited greater difficulties in emotion regulation, reflected by higher total DERS-16 scores (p<0.001). Anxiety and depressive symptom severity, measured using the HADS, were significantly higher in the IGM group for both anxiety, HADS-A, and depression, HADS-D, subscales (both p<0.001). The effect sizes confirmed clinically meaningful between-group differences (Table 2).

**Table 2:** Psychological scale scores between groups.

Scale/subscale	IGM (n=97) mean ± SD	Control (n=00) mean ± SD	Mean difference	t	p-value	Effect size (Cohen’s d)
TEMPS-depressive	18.99±2.20	16.00±0.82	2.99	12.713	<0.001	1.81
TEMPS-anxious	20.92±1.71	17.00±0.82	3.92	20.657	<0.001	2.94
TEMPS-cyclothymic	18.03±1.23	17.44±0.49	0.59	4.447	<0.001	0.63
TEMPS-hyperthymic	12.88±1.19	13.56±0.49	-0.68	-5.277	<0.001	-0.75
TEMPS-irritable	15.68±1.27	14.89±0.73	0.79	5.359	<0.001	0.76
DERS-16 total	43.27±3.48	36.78±3.15	6.49	17.418	<0.001	2.48
HADS-A (anxiety)	11.69±1.31	8.84±0.69	2.85	19.269	<0.001	2.10
HADS-D (depression)	9.61±1.29	7.55±0.50	2.06	14.793	<0.001	2.07

Values are mean±SD. Negative values indicate lower scores in the IGM group for the hyperthymic temperament subscale. Effect sizes are Cohen’s d; 0.20=small, 0.50=medium, ≥0.80=large. IGM: idiopathic granulomatous mastitis; SD: standard deviation; TEMPS: Temperament Evaluation of Memphis, Pisa, Paris, and San Diego; DERS-16: Difficulties in Emotion Regulation Scale; HADS: Hospital Anxiety and Depression Scale; HADS-A: Hospital Anxiety and Depression Scale-Anxiety; HADS-D: Hospital Anxiety and Depression Scale-Depression.

### Distribution of dominant affective temperaments

The distribution of dominant affective temperament types differed significantly between the IGM and control groups (χ^2^=21.95, df=5, p<0.001; Cramér’s V=0.334). Depressive and anxious dominant temperaments were more prevalent among women with IGM, whereas hyperthymic and irritable dominant temperaments were more frequent in the control group. A calm temperament was rare and observed only in the IGM group.

### Recurrence after initial treatment

A multivariable binary logistic regression analysis examining predictors of recurrence did not yield a statistically significant overall model (p=0.127). However, anxious temperament emerged as a significant independent predictor, with lower TEMPS-anx scores associated with a higher likelihood of recurrence (OR 0.33, 95%CI 0.14–0.78, p=0.012). The emotion regulation difficulties (DERS-16) showed a trend toward significance (p=0.057), whereas depressive temperament and HADS anxiety–depression categories were not significantly associated with recurrence.

### Treatment resistance

The psychological variables significantly predicted treatment resistance in women with IGM. The multivariable logistic regression model was statistically significant (χ^2^=13.48, p=0.019) and demonstrated acceptable explanatory power (Nagelkerke R^2^=0.173) with good model fit (Hosmer-Lemeshow p=0.722). Higher levels of emotion regulation impairment (DERS-16) were strongly associated with treatment resistance (OR 2.86, 95%CI 1.41–5.80, p=0.004). In contrast, anxious temperament was inversely associated with treatment resistance, indicating a protective effect (OR 0.35, 95%CI 0.15–0.78, p=0.010). Participants classified as having elevated anxiety–depression risk based on HADS scores (8–21) were also less likely to exhibit treatment resistance compared with those in the normal range (OR 0.18, 95%CI 0.04–0.81, p=0.025). Depressive temperament was not significantly associated with treatment resistance. Detailed regression results are presented in Table 3.

**Table 3:** Multivariable logistic regression analysis predicting treatment resistance in women with idiopathic granulomatous mastitis.

Variables	B	SE	Wald	P	OR [Exp(B)]	95%CI (lower)	95%CI (upper)
TEMPS-depressive	0.079	0.220	0.128	0.720	1.08	0.70	1.67
TEMPS-anxious	-1.063	0.415	6.563	0.010	0.35	0.15	0.78
DERS-16 Total	1.051	0.361	8.498	0.004	2.86	1.41	5.80
HADS category (0–7 vs. 8–21)	-1.703	0.762	4.999	0.025	0.18	0.04	0.81
Constant	-4.999	4.136	1.461	0.227	–	–	–

Multivariable binary logistic regression results. OR: odds ratio; CI: confidence interval; SE: standard error; TEMPS: Temperament Evaluation of Memphis, Pisa, Paris, and San Diego; DERS-16: Difficulties in Emotion Regulation Scale; HADS: Hospital Anxiety and Depression Scale. HADS category: 0–7=normal, 8–21=borderline or clinical case.

### Fistula formation

The logistic regression analysis evaluating predictors of fistula formation did not identify any significant psychological predictors. None of the affective temperament dimensions, emotion regulation scores, or anxiety–depression categories were associated with fistula development (all p>0.20).

## DISCUSSION

This controlled case–control study demonstrates that IGM is associated with a distinct psychosomatic vulnerability profile characterized by elevated depressive and anxious temperament traits and marked impairments in emotion regulation. Beyond documenting the psychological distress, these findings suggest that stable affective dispositions and emotion regulation capacity may be clinically relevant factors influencing treatment response in IGM. These results extend psychoneuroim-munological models by supporting a link between persistent emotional styles and inflammatory disease outcomes in a condition where biological mechanisms alone do not fully explain clinical heterogeneity^6,7,8,9^.

### Emotion regulation and treatment resistance

The most robust finding of this study is the strong association between impaired emotion regulation and treatment resistance. Higher DERS-16 scores independently predicted failure to respond to corticosteroid-based therapy, even after adjustment for affective temperament traits and anxiety–depression symptom burden. Although the present analysis focused on total DERS-16 scores, future studies incorporating subscale-level analyses and biological markers may clarify which specific facets of emotion dysregulation most strongly influence treatment resistance in IGM^13^.

### Anxious temperament and the vigilance hypothesis

An unexpected but clinically meaningful finding was the inverse association between anxious temperament and both recurrence and treatment resistance. While acute anxiety states are often linked to heightened inflammatory responses, trait anxiety represents a more stable, vigilance-oriented emotional disposition rather than transient stress reactivity^6,7,8,9^. This pattern aligns with the “vigilance hypothesis,” whereby moderate trait anxiety enhances symptom awareness, healthcare engagement, and treatment adherence without provoking maladaptive stress responses^14,15,16^. Of note, this interpretation is consistent with psychobiological frameworks such as Leventhal’s Common-Sense Model of illness self-regulation, which emphasizes the role of cognitive–emotional illness representations in shaping monitoring, adherence, and coping behaviours^14^. If replicated, these findings suggest that interventions aimed at fostering adaptive vigilance—through psychoeducation, cognitive–behavioural strategies, or illness-perception–focused approaches—may improve clinical outcomes in women with IGM^16^.

### Systemic versus local disease processes

In contrast to recurrence and treatment resistance, none of the psychological variables predicted fistula formation, which suggests that these outcomes reflect distinct biological processes. Recurrence and treatment resistance likely represent manifestations of systemic psychoneuroimmune susceptibility mediated by neuroendocrine and immune pathways, whereas fistula formation appears to be driven primarily by local mechanical and tissue-destructive factors, including abscess pressure, ductal anatomy, and focal macrophage activation. As such, similar patterns have been reported in inflammatory bowel disease and psoriasis, where psychological factors influence disease activity but do not predict structural complications^10,11,12^.

### Clinical implications

Taken together, these findings support the clinical relevance of psychoneuroimmunology-informed psychological assessment in IGM. Screening for emotion regulation difficulties and affective temperament traits may help identify patients at increased risk of treatment resistance and guide more individualized therapeutic strategies. Importantly, these assessments are low-cost, non-invasive, and feasible within routine clinical practice. As a matter of fact, depression in the elderly and women has its own health consequences^17,18^, and also IGM is a crucial area in the field of breast diseases for mastology health providers^19,20,21,22,23^.

### Strengths and limitations

The strengths of this study include its controlled design, histopathological confirmation of IGM, use of validated psychometric instruments, and focus on clinically meaningful outcomes. Nevertheless, several limitations should also be acknowledged. The cross-sectional design precludes causal inference, and the single-center sample may limit generalisability. Furthermore, the absence of biological biomarkers prevents direct testing of the proposed psychoneuroendocrine mechanisms. In addition, the exclusion of patients receiving psychiatric medication may have reduced variability in psychological profiles. Finally, logistic regression models with modest event counts should be interpreted cautiously.

## CONCLUSION

In essence, IGM, per se, is associated with a distinct psychosomatic vulnerability profile. Impaired emotion regulation appears to be a clinically relevant risk factor for treatment resistance, whereas moderate levels of anxious temperament may exert protective effects by promoting vigilant health behaviors and treatment adherence among providers. Frankly, these findings underscore the importance of integrating psychoneuroimmu-nology-informed psychological assessment into the clinical management of IGM. Ultimately, routine screening of temperament traits and emotion regulation capacity may support more individualized treatment strategies and contribute to improved clinical outcomes in mastology.

## Data Availability

The datasets generated and/or analyzed during the current study are available from the corresponding author upon reasonable request.
